# Endoplasmic reticulum KDEL-tailed cysteine endopeptidase 1 of *Arabidopsis* (AtCEP1) is involved in pathogen defense

**DOI:** 10.3389/fpls.2014.00058

**Published:** 2014-02-24

**Authors:** Timo Höwing, Christina Huesmann, Caroline Hoefle, Marie-Kristin Nagel, Erika Isono, Ralph Hückelhoven, Christine Gietl

**Affiliations:** ^1^Lehrstuhl für Botanik, Center of Life and Food Sciences Weihenstephan, Technische Universität MünchenFreising, Germany; ^2^Lehrstuhl für Phytopathologie, Center of Life and Food Sciences Weihenstephan, Technische Universität MünchenFreising, Germany; ^3^Department of Plant Systems Biology, Center of Life and Food Sciences Weihenstephan, Technische Universität MünchenFreising, Germany

**Keywords:** programmed cell death, plant immunity, sporulation, haustorium, cell wall

## Abstract

Programmed cell death (PCD) is a genetically determined process in all multicellular organisms. Plant PCD is effected by a unique group of papain-type cysteine endopeptidases (CysEP) with a C-terminal KDEL endoplasmic reticulum (ER) retention signal (KDEL CysEP). KDEL CysEPs can be stored as pro-enzymes in ER-derived endomembrane compartments and are released as mature CysEPs in the final stages of organelle disintegration. KDEL CysEPs accept a wide variety of amino acids at the active site, including the glycosylated hydroxyprolines of the extensins that form the basic scaffold of the cell wall. In *Arabidopsis*, three KDEL CysEPs (*AtCEP1*, *AtCEP2*, and *AtCEP3*) are expressed. Cell- and tissue-specific activities of these three genes suggest that KDEL CysEPs participate in the abscission of flower organs and in the collapse of tissues in the final stage of PCD as well as in developmental tissue remodeling. We observed that *AtCEP1* is expressed in response to biotic stress stimuli in the leaf. *atcep1* knockout mutants showed enhanced susceptibility to powdery mildew caused by the biotrophic ascomycete *Erysiphe cruciferarum*. A translational fusion protein of AtCEP1 with a three-fold hemaglutinin-tag and the green fluorescent protein under control of the endogenous AtCEP1 promoter (P_CEP1_::pre-pro-3xHA-EGFP-AtCEP1-KDEL) rescued the pathogenesis phenotype demonstrating the function of AtCEP1 in restriction of powdery mildew. The spatiotemporal *AtCEP1*-reporter expression during fungal infection together with microscopic inspection of the interaction phenotype suggested a function of AtCEP1 in controlling late stages of compatible interaction including late epidermal cell death. Additionally, expression of stress response genes appeared to be deregulated in the interaction of *atcep1* mutants and *E. cruciferarum*. Possible functions of AtCEP1 in restricting parasitic success of the obligate biotrophic powdery mildew fungus are discussed.

## Introduction

Programmed cell death (PCD) is a genetically determined, highly regulated process in all multicellular organisms (Hadfield and Bennett, 1997). PCD causes the loss of unpollinated ovules and the collapse of nucellus cells; it eliminates tissues and cells serving temporary functions during development such as the tapetum cells in anthers, the elimination of suspensor cells connecting the embryo to the mother plant and dissolution of endosperm cells in germinating castor beans (Pennel and Lamb, 1997; Olsen et al., 1999; Young and Gallie, 2000). Plants furthermore limit the spread of fungal or bacterial pathogens by rapid cell death at the site of infection through a mechanism called the hypersensitive response (HR) (Dickman and Fluhr, 2013).

Diverse classes of proteases are involved in PCD, including cysteine proteases, serine proteases, aspartic proteases and metalloproteases (Beers, 1997; Beers et al., 2000, 2004; Schaller, 2004). In plant PCD, special functions are described for vacuolar proteases (Müntz, 2007; Hara-Nishimura and Hatsugai, 2011), metacaspases (Lam and del Pozo, 2000; Xu and Zhang, 2009; Tsiatsiani et al., 2011) or subtilisin-like proteases (Vartapetian et al., 2011). Specific for plant PCD is a unique group of papain-type cysteine endopeptidases (CysEPs) characterized by a C-terminal KDEL endoplasmic reticulum (ER) retention signal (KDEL CysEPs) with RcCysEP from castor bean (*Ricinus communis*) as the founding member (Schmid et al., 1998). KDEL CysEPs are not present in mammals or fungi, but are ubiquitous in plants (Hierl et al., 2012).

KDEL CysEPs exhibit a characteristic and unusually broad substrate specificity. The cleavage site ↓ within a substrate is denoted as P2-P1-↓-P1′-P2′. KDEL CysEPs have a clear preference for neutral amino acids with large aliphatic and non-polar (Leu, Val, Met) or aromatic (Phe, Tyr, Trp) side-chains in the P2 position and no clear preference in the P1 position, as it is typical for papain-type CysEPs. Unusually, they accept proline in the P1 and P1′ positions (Than et al., 2004; Hierl et al., 2013). Crystallization of the purified RcCysEP from castor bean as the founding member of KDEL CysEPs (Schmid et al., 1998) revealed that castor bean CysEP folds into two distinct domains of roughly equal size, as it is usual for papain-like CysEPs. The folding of RcCysEP is also very similar to the proline-specific cysteine peptidase from ginger (*Zingiber officinale*). The active site cleft of RcCysEP, however, is wider when compared to the ginger protease and papain (Than et al., 2004). RcCysEP can therefore digest extensins with its ability to accept glycosylated hydroxy-proline near the cleavage site (Helm et al., 2008). The respective amino acids (Leu, Met, Ala, Leu, Asn, Gly), which are decisive for this generally more open appearance of the active site cleft, together with the amino acids defining the catalytic pocket (Cys, His, Gln, Asn), are highly conserved among all known KDEL CysEPs (Hierl et al., 2012). Possibly, all KDEL CysEPs share the same broad substrate specificity. Extensins build the basic scaffold of the plant cell wall (Cannon et al., 2008), and thus KDEL CysEPs might support final cell collapse. KDEL CysEPs seem to have a dual set of substrates: digesting cytoplasmic components in cells of dying tissues for recycling to the surviving parts of the plant or in cells of germinating seedlings for mobilization of storage proteins, respectively, and digesting cell wall extensins in the final stage of PCD in support of the general cell collapse for tissue break down. Furthermore, KDEL CysEPs are expressed during tissue remodeling, possibly for clearance of dead cells and for generating space for plant organ outgrowth (Helm et al., 2008; Hierl et al., 2013).

It is obvious that KDEL-CysEPs are found in tissues undergoing PCD, especially in cells that finally collapse, such as the hypogeous cotyledons of *Vicia sativa* (Becker et al., 1997), the maturing pods of *Phaseolus vulgaris* (Tanaka et al., 1991), the unpollinated ovaries of *Pisum sativum* (Cercos et al., 1999), the outer integument developing into the seed coat of *Phalaenopsis* (Nadeau et al., 1996), the senescing flower petals of *Hemerocallis* (Valpuesta et al., 1995) and *Sandersonia aurantiaca* (O'Donghue et al., 2002), the megagametophyte cells after germination of *Picea glauca* seeds (He and Kermode, 2003), and the epigeous cotyledons of *Vigna mungo* (Toyooka et al., 2000). KDEL CysEPs were found in the senescing endosperm of germinating castor bean seeds (Schmid et al., 1999, 2001) and in the nucellus in maturing castor bean seeds, where the endosperm expands at the expense of the nucellus cells (Greenwood et al., 2005). They are expressed in both developing and dehiscing tomato anthers (*Solanum lycopersicum*) (Senatore et al., 2009) and in endosperm cells of imbibed tomato seeds (Trobacher et al., 2013).

In *Arabidopsis*, three KDEL CysEPs: AtCEP1 (At5g50260), AtCEP2 (At3g48340), and AtCEP3 (At3g48350) have been identified that are expressed in tissues undergoing PCD. Determination of promoter activities using β-glucuronidase as reporter in *Arabidopsis* transformants elucidated a remarkable tissue- and organ-specificity: *AtCEP1* and *AtCEP3* promoter activities were found in generative tissues at several stages of seed and fruit development such as *AtCEP1* in the abscission zone and the nectaries of a silique or *AtCEP3* in the maturing carpels. *AtCEP1*, *AtCEP2*, and *AtCEP3* promoter activities were found in vegetative tissue such as *AtCEP1* in the course of lateral root formation, *AtCEP2* in roots within the root elongation zone and the beginning root cap, and *AtCEP3* at the hypocotyl-root transition zone or in trichomes of leaves (Helm et al., 2008; Hierl et al., 2013).

KDEL CysEP are synthesized as pre-pro-enzymes and are co-translationally transferred into the ER, where the pre-sequence is removed. KDEL CysEPs can be stored as enzymatically inactive pro-enzymes in ER-derived compartments.

A spherical organelle surrounded by a single ribosome-studded membrane with a diameter averaging 1 μm was found in senescing endosperm tissue from castor bean. This organelle was discovered in ultrastructural and cytochemical studies independently by two groups in 1970. It was called “dilated cisternae,” since it seemed to develop from the ER (Vigil, 1970), or “ricinosome,” since it was found only in castor bean at that time (Mollenhauer and Totten, 1970). The ricinosomes were “re-discovered” with the identification of their marker enzyme, the KDEL CysEP (Schmid et al., 1998). Ricinosomes with their KDEL CysEP have been identified by immuno-electron-microscopy in the endosperm of germinating castor bean seeds (Schmid et al., 1999, 2001), in the nucellus of maturing castor bean seeds (Greenwood et al., 2005), in flower petals of *Hemerocallis* (Schmid et al., 1999), in the cotyledons of *Vicia sativa* (Becker et al., 1997), the unpollinated ovaries of *Pisum sativum* (Cercos et al., 1999), in tomato anthers (Senatore et al., 2009) and in endosperm cells of tomato seeds (*Solanum lycopersicum*) (Trobacher et al., 2013). Hence, the accumulation of KDEL CysEPs and the appearance of ricinosomes may be used as an early predictor of PCD.

KDEL-tailed protease-accumulating vesicles (KDEL vesicles, KVs) in germinating mung bean (*Vigna mungo*) cotyledons are similar to ricinosomes in that they accumulate the KDEL-tailed cysteine protease SH-EP (Toyooka et al., 2000). In contrast to ricinosomes, immunocytochemistry identified KDEL vesicles to transport large amounts of SH-EP from the endoplasmic reticulum to protein storage vacuoles. The mass transport of the proteinase by KDEL vesicles is thus involved in the protein mobilization of plants (Toyooka et al., 2000; Okamoto et al., 2003).

Interestingly, two different types of ER-derived organelles were found in *Arabidopsis* seedlings for storage of KDEL CysEPs using the mCherry-AtCEP2 reporter fusion protein (Hierl et al., 2013). mCherry-AtCEP2 was detected in the epidermal layers of leaves, hypocotyls and roots; in the root, it was predominantly found in the elongation zone and root cap. Co-localization with an ER membrane marker showed that mCherry-AtCEP2 was stored in 10 μm long spindle shaped organelles as well as round vesicles with a diameter of approximately 1 μm. The long organelles appear to be ER bodies, which are found specifically in Brassicales. The round vesicles strongly resemble ricinosomes (Hierl et al., 2013).

In plant microbe-interaction PCD has to be tightly controlled. Biotrophic pathogens are restricted by PCD because they strictly depend on living host tissue to feed from. PCD is an integral part of the HR by which plants restrict biotrophs in particular if triggered by recognition of microbial effectors. By contrast, if host PCD is triggered by hemibiotrophic or necrotrophic pathogens, it may foster disease by producing dead defenseless tissue that is easily accessible for the pathogen (Dickman and Fluhr, 2013). Papain-type cysteine proteases are involved in plant-microbe interactions. They are expressed in response to biotic stress and can be direct or indirect targets of microbial virulence effectors (Shindo and Van der Hoorn, 2008). Publicly available expression data (www.genevestigator.com; Zimmermann et al., 2004) suggested that *AtCEP1* (At5g50260, probe set ID 248545_at) is expressed in hormone response such as auxin in mutants of the constitutive photomorphogenic9 signalosome (*csn4 and csn3*; Dohmann et al., 2008) and in mutants that constitutively express defense responses such as *cpr5* (Bowling et al., 1997; Clarke et al., 2000). *AtCEP3* (At3g48350; probe set ID 252365_at) does not exhibit such a pronounced response, and no expression data are available for *AtCEP2* (At3g48340). We hence wanted to know, if AtCEP1 is involved in pathogen defense. Therefore, we chose the interaction with an obligate biotrophic powdery mildew fungus *Erysiphe cruciferarum* because it allows for observation of quantitative disease phenotypes. We further had observed that a certain degree of late epidermal cell death occurred in the interaction of *Arabidopsis* with *E. cruciferarum* and thus analyzed wild type *AtCEP1* and *atcep1* mutant phenotypes in this interaction. Data introduce a function for AtCEP1 in limiting susceptibility of *Arabidopsis* to *E. cruciferarum* and suggest a role in controlling late stages of the compatible interaction. Apparently, *AtCEP1*-dependent PCD at late stages of the compatible interaction fulfills a function in limiting parasitic growth of the fungus.

## Materials and methods

### Generation of reporter lines expressing pre-pro-3xHA-EGFP-AtCEP1-KDEL and pre-pro-3xHA-EGFP-KDEL, respectively, under control of the endogenous AtCEP1 promoter in the *atcep1* knockout mutant

For the cloning strategy of the fusion gene coding for pre-pro-3xHA-EGFP-AtCEP1-KDEL under the control of the endogenous promoter of *AtCEP1* (P_CEP1_::pre-pro-3xHA-EGFP-AtCEP1-KDEL) and the primers used see Supplemental Figure S1. The sequence approximately 2000 bp upstream of the start Met, that was previously shown to confer tissue specific expression (Helm et al., 2008), was used as the *AtCEP1* promoter region. We placed the first three amino acids Leu-Pro-Thr of the mature subunit C-terminal to the pro-sequence in front of the 3xHA tag in order to ensure processing of the pro-peptide during maturation of AtCEP1. The *AtCEP1* promoter with the adjacent 5'UTR and the coding region for the pre-pro-sequence were amplified from WT (Col0) genomic DNA isolated by cetyl-trimethyl-ammonium bromide (CTAB) extraction (Murray and Thompson, 1980). The 3xHA tag was amplified from pNIGEL18 (Geldner et al., 2009) and EGFP was amplified from pEZS-CL (Cutler et al., 2000). The mature AtCEP1 subunit with the 3'UTR was amplified from WT (Col0) genomic DNA. The resulting PCR products were cloned into pGREEN conferring kanamycin resistance (Hellens et al., 2000; www.ac.uk). The final plasmid construct was sequenced and transformed into *Agrobacterium tumefaciens* (pGV3101) by electroporation. The construct P_CEP1_::pre-pro-3xHA-EGFP-KDEL as a non-functional reporter protein lacking the mature *AtCEP1* protease subunit was obtained in an analogous manner. It comprised the endogenous AtCEP1 promoter, the 5′UTR and the coding regions for the N-terminal pre-pro-peptide, for the 3xHA tag and EGFP and for the nine C-terminal amino acids of the mature AtCEP1 subunit, including the ER retention signal KDEL (for cloning strategy and primers used see Supplemental Figure S2). The resulting PCR products were cloned into pGREEN (Hellens et al., 2000). The final plasmid construct was sequenced and transformed into *Agrobacterium tumefaciens* (pGV3101) by electroporation.

Flowers from homozygous *atcep1* knock out mutant plants (SAIL_158_B06) were transformed by floral dipping (Clough and Bent, 1998) resulting in plants expressing the functional (P_CEP1_::pre-pro-3xHA-EGFP-AtCEP1-KDEL) or non-functional (P_CEP1_::pre-pro-3xHA-EGFP-KDEL) EGFP-reporter proteins, respectively. Eight different homozygous transformants for each construct were screened for high expression of the fusion protein by confocal laser scanning microscopy (CLSM) and three were chosen for further analysis.

### Leaf infection with powdery mildew and symptoms rating

*Arabidopsis thaliana* Col0 plants and the *AtCEP1* T-DNA insertion mutants (SAIL_158_B06 and SALK_013036) as well as *atcep1* knock out plants transformed with the functional reporter (P_CEP1_::pre-pro-3xHA-EGFP-AtCEP1-KDEL in SAIL_158_B06) and *atcep1* knock out plants transformed with the non-functional reporter (P_CEP1_::pre-pro-3xHA-EGFP-KDEL in SAIL_158_B06) were grown in a growth chamber at 22°C and a 10-h photoperiod with 120 μmol m^−2^ s^−2^ light and 65% relative humidity. The *Arabidopsis* compatible powdery mildew fungus *Erysiphe cruciferarum* was grown on Arabidopsis Col-0 plants and for increased conidia production on Arabidopsis pad4 mutant plants (Glazebrook et al., 1996) at the same conditions. Five-week-old *Arabidopsis* plants were inoculated with *E. cruciferarum* for macroscopy and microscopy evaluation of disease progression with a density of 5–7 conidia mm^−2^. *Arabidopsis* susceptibility to *E. cruciferarum* was scored by visual examination of the whole plant 7, 9, 11, and 13 d after inoculation. Plants were distributed in three categories of susceptibility with 0–30%, 30–60%, and >60% diseased leaf area.

### Staining of fungal structures with wheat germ agglutinine-tetramethylrhodamine

Wheat germ agglutinin tetramethylrhodamin conjugate (WGA-TMR, Invitrogen Molecular Probes, Germany) binds to extra- and intracellular chitin of fungi. To investigate the development of *E. cruciferarum* on *Arabidopsis*, inoculated leaves were harvested 5 days after inoculation and were discolored in ethanol-acetic acid glacial (EtOH-HAc; 6:1). Before WGA-TMR staining, the leaves were washed with H_2_O_dest_ to remove the EtOH-HAc solution and were incubated for 6 min in PBS buffer (140 mM NaCl, 2.7 mM KCl, 4.3 mM Na_2_HPO_4_, 1.5 mM KH_2_PO4, pH7.4). The leaves were placed into the staining solution [0.01 μ g/μ l WGA-TMR (Molecular Probes, Invitrogen), 0.01 μ g/μ l BSA, PBS buffer] and were vacuum infiltrated twice at −0,8 bar. After 24–48 h incubation in the dark at 4°C, three leaves from WT or *atcep1* plants each containing 54 colonies, respectively, were analyzed by fluorescence microscopy (Olympus BX61TRF, Japan). The results were reproduced in a second independent inoculation with 48 colonies analyzed on three leaves of WT and *atcep1* plants, respectively.

### Callose staining with methyl blue

Callose depositions in *Arabidopsis* cells were visualized by methyl blue (Sigma Aldrich Chemie GmbH, München, Germany) staining. The discolored and WGA-TMR stained leaves were rinsed with distilled water and transferred into 67 mM K_2_HPO_4_ buffer for 10 min followed by incubation for 3–4 h in the staining solution (0.05% methyl blue in 67 mM K_2_HPO_4_) in the dark and direct analysis by fluorescence microscope (Olympus BX61TRF, Japan).

### qRT-PCR

Primers used for qRT-PCR are: ACT8 qRT fw: TGAGACCTTTAATTCTCCAGCTATG; ACT8 qRT rv: CCAGAGTCCAACACAATACCG; PR1 qRT fw: GATGTGCCAAAGTGAGGTGTAA; PR1 qRT rv: TTCACATAATTCCCACGAGGA; PDF1.2 qRT fw: GTTCTCTTTGCTGCTTTCGAC; PDF1.2 qRT rv: GCAAACCCCTGACCATGT. Total RNA was extracted from leaves before and 12 h (hpi) and 1, 2, 3, and 5 days (dpi) post mildew inoculation. Leaves were collected from at least five individual plants and total RNA was extracted with a NucleoSpin RNA plant kit (Machery-Nagel) and 2 μg of total RNA was reverse-transcribed with an oligo-dT primer and M-MulV Reverse Transcriptase (Fermentas) following the manufacturers' instructions. Quantitive real-time PCR was performed using iQ SYBR Green Supermix (Bio-Rad) in a CFX96 Real-Time System Cycler (Bio-Rad). A 50-cycle two-step amplification protocol (10 s at 95°C, 25 s at 60°C) was used for all measurements.

### Biochemical methods

Leaves were harvested 0–18 dpi days post inoculation with powdery mildew. A protein extract was prepared from one leaf (20–30 mg fresh weight). The plant material was ground with mortar and pestle under liquid N_2_ followed by the addition of loading dye (60 mM Tris-HCl pH 6.6, 5% glycerine, 1.5% SDS, 1.5% β-ME, 0.1% bromophenol blue final concentration), incubation for 10 min/95°C and analysation by SDS-PAGE followed by western blot analysis with anti-HA antibodies (Roche).

### Confocal laser scanning microscopy

CLSM (Fluoview FV 1000, Olympus, Japan) was performed using excitation at 488nm and emission detection between 503 and 550 nm for GFP. Single pictures or stacks of pictures with 0.5 μm increments at higher resolution and 2.5 μm increments at lower resolution were made.

## Results

### Homozygous *atcep1* knock out mutants exhibit enhanced susceptibility to the biotrophic fungus *erysiphe cruciferarum*

**Figure 1:**
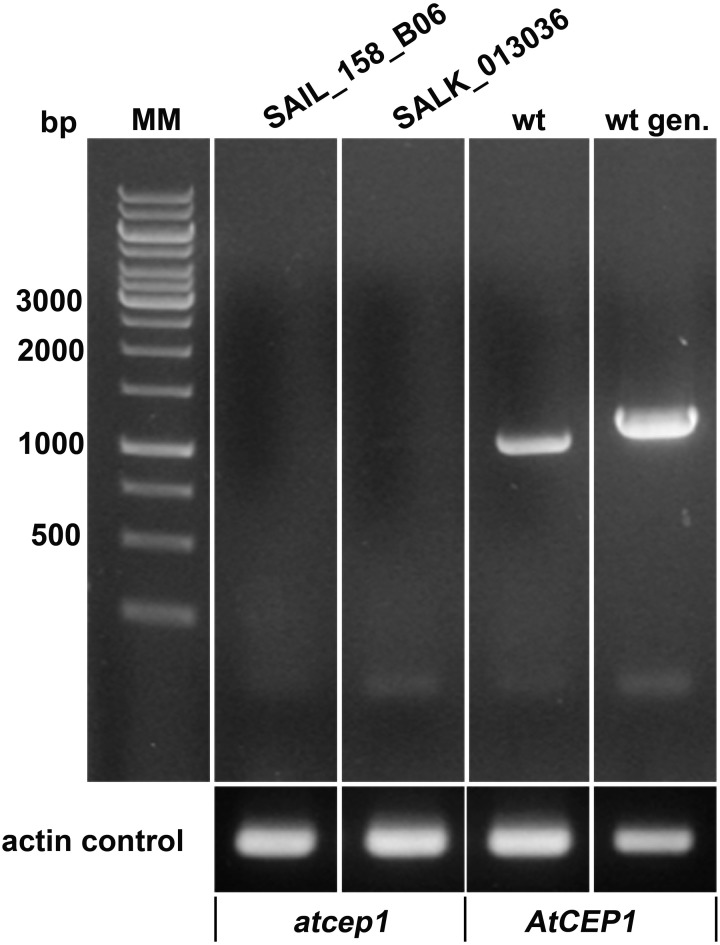
**Both AtCEP1 mutants SAIL_158_B06 and SALK_013036, harboring the T-DNA insertion in the 3. exon represents loss of function mutants**. No corresponding transcript could be amplified by RT-PCR using primers that comprise the complete coding region from 7 days old seedlings, whereas the parent Col-0 wild type expressed the gene. wt, RT-PCR on wild type RNA; wt gen, PCR on wild type genomic DNA.

**Figure 2:**
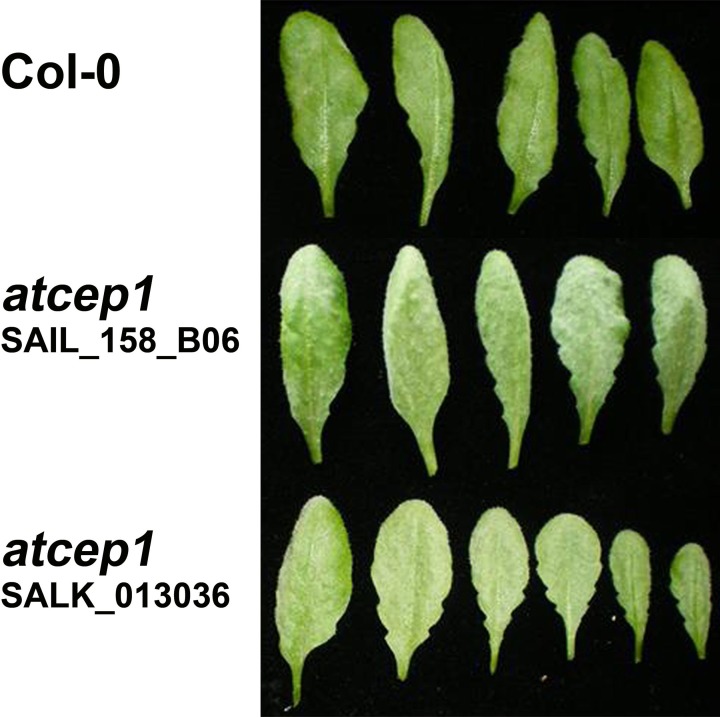
**Homozygous *atcep1* knock out mutants with a T-DNA insertion in the 3. Exon exhibit enhanced susceptibility upon leaf inoculation with the biotrophic fungus *Erysiphe cruciferarum***. Leaves are shown 11 days post low density inoculation.

Two *AtCEP1* insertion lines, SAIL_158_B06 and SALK_013036, have the T-DNA insertion within the 3rd Exon. Both lines were homozygous, and RT-PCR using primers that amplified the coding region (spanning the T-DNA) confirmed the knock out of *AtCEP1* (Figure 1). Wild type Col0 plants and the two homozygous *atcep1* knock out mutants were inoculated with conidia of the powdery mildew fungus *E. cruciferarum*, an obligate biotroph that requires living cells for growth. Five week old plants were inoculated for evaluation of disease progression. *Arabidopsis* susceptibility to *E. cruciferarum* was scored by visual examination of the whole plant 7, 9, 11, and 13 days after inoculation. Both independent *atcep1* knock out mutants lines exhibited the same phenotype, that is enhanced susceptibility to *E. cruciferarum* as compared to the parental wild type, as can be seen by visual examination of the whole plant for scoring the leaf area covered by powdery mildew symptoms (Figure 2, Supplemental Figure S3, see also below).

### A functional reporter construct complements the pathogenesis phenotype of *atcep1* knock out

**Figure 3:**
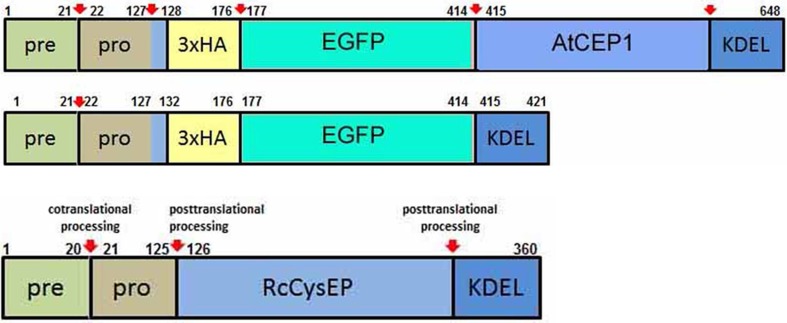
**Schematic representation of the functional reporter protein pre-pro-3xHA-EGFP-AtCEP1-KDEL and the non-functional reporter protein pre-pro-3xHA-EGFP-KDEL expressed under the control of the endogenous AtCEP1 promoter**. Both constructs were transformed into *atcep1* knock out mutant line SAIL_158_B06. Pre-pro-RcCysEP is shown for comparison. Red arrows indicate proven or predicted protein cleavage sites.

We constructed the fusion genes under the control of the endogenous *AtCEP1* promoter for functional (P_CEP1_::pre-pro-3xHA-EGFP-AtCEP1-KDEL) and non-functional (P_CEP1_::pre-pro-3xHA-EGFP-KDEL) reporter constructs including EGFP with and without the mature AtCEP1 subunit (Figure 3, Supplemental Figures S1, S2, see Materials and Methods). The final plasmid constructs were sequenced and transformed into *Agrobacterium tumefaciens* for subsequent transformation into *atcep1* knock out plants SAIL_158_B06 in order to obtain plants expressing the functional or non-functional reporter proteins, respectively. Eight homozygous transformants for each construct were obtained. Three transformants for each construct exhibiting the highest fluorescence were chosen for further pathogen inoculation.

**Figure 4:**
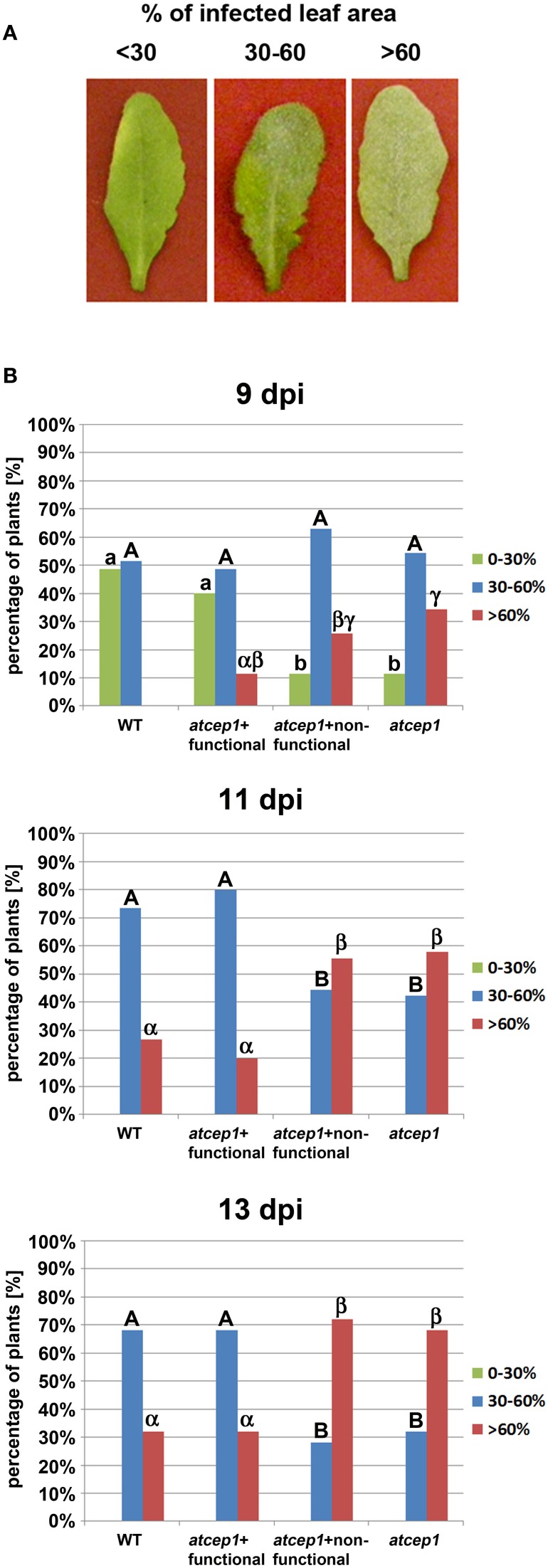
**Disease symptoms of wild type and *atcep1* mutant plants (SAIL_158_B06) upon leaf infection with *Erysiphe cruciferarum* spores**. Wild type Col0 plants and *atcep1* (SAIL_158_B06) plants transformed with the functional construct P_CEP1_::pre-pro-3xHA-EGFP-AtCEP1-KDEL that are comparable to wild type plants, and *atcep1* mutant plants (SAIL_158_B06) transformed with the non-functional reporter P_CEP1_::pre-pro-3xHA-EGFP-KDEL that are comparable to *atcep1* knock out plants were infected with *E. cruciferarum* and disease symptoms were scored after visual inspection of the whole plant 9, 11, and 13 days post inoculation (dpi). Infected leaf were distributed in the three categories <30%, 30–60%, and >60% diseased leaf area. **(A)** Representative leaves were excised and photographed 11 dpi. **(B)** Columns marked with different letters indicate statistically different groups according to the ANOVA- and Duncan test (*p* < 0.05) and represent the frequency of plants distributed in the three categories of susceptibility. Data represent the respective means of seven experiments from independent inoculation events of the mutants with the corresponding parent background control. Each experiment comprised 5 plants per line.

Five week old plants were inoculated with the powdery mildew fungus *E. cruciferarum* and were scored for disease progression by visual examination of the whole plant 9, 11, and 13 days post inoculation: Plants were distributed in the three categories of susceptibility with less than 30%, 30–60%, and more than 60% of infected leaf area (Figure 4A). Significantly more plants of the *atcep1* knock out line SAIL_158_B06 were classified in the category with >60% diseased leaf area compared to Col0 control plants. This was particularly obvious at later stages of the interaction, resulting in a super-susceptibility phenotype of the mutants. By contrast, *atcep1* knock out plants transformed with the functional reporter construct reporter P_CEP1_::pre-pro-3xHA-EGFP-AtCEP1-KDEL exhibited a similar basal resistance to *E. cruciferarum* as compared to wild type Col0 plants thus proving the functionality of the reporter protein and complementation of the mutant phenotype. On the other hand, *atcep1* knock out plants transformed with the non-functional reporter construct reporter P_CEP1_::pre-pro-3xHA-EGFP-KDEL behaved similar to the original *atcep1* knock out plants in showing super-susceptibility (Figure 4B).

### EGFP-AtCEP1 expression correlates with the appearance of leaf symptoms during infection with powdery mildew

**Figure 5:**
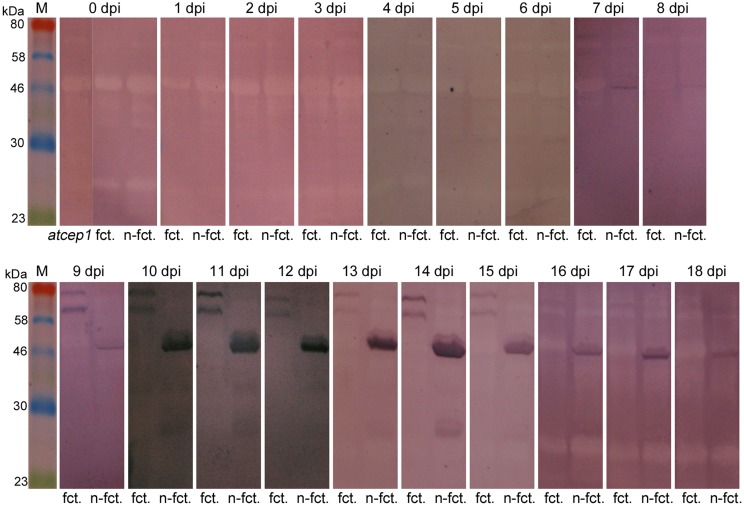
**Pro-3xHA-EGFP-AtCEP1-KDEL and pro-3xHA-EGFP-KDEL, respectively, are detectable from 9 days post inoculation (dpi) on upon leaf inoculation with *Erysiphe cruciferarum***. Immunoblots with anti-HA antibodies on total protein extracts from leaves of “wild type” plants (*atcep1* SAIL_158_B06 transformed with the functional construct P_CEP1_::pre-pro-3xHA-EGFP-AtCEP1-KDEL) and *atcep1* knock out plants (SAIL_158_B06 and SAIL_158_B06 transformed with the non-functional construct P_CEP1_::pre-pro-3xHA-EGFP-KDEL). *atcep1*, SAIL_158_B06; fct, *atcep1* plants transformed with the functional reporter pre-pro-3xHA-EGFP-AtCEP1-KDEL; n-fct, *atcep1* plants transformed with the non-functional reporter pre-pro-3xHA-EGFP-KDEL.

For detection of AtCEP1 protein expression, we scored the appearance of AtCEP1 on total protein extracts from infected leaves of *atcep1* knock out plants transformed with the functional reporter P_CEP1_::pre-pro-3xHA-EGFP-AtCEP1-KDEL that are comparable to wild type Col0 plants and *atcep1* knock out plants transformed with the non-functional reporter P_CEP1_::pre-pro-3xHA-EGFP-KDEL that are comparable to *atcep1* knock out plants by immunoblot analysis with anti-HA antibodies (Figure 5). Pro-3xHA-EGFP-AtCEP1-KDEL was detectable from day 9 on post inoculation and disappeared after day 15. Typically, two distinct protein bands are recognized by the anti-HA antibodies. Both proteins exhibit with molecular masses smaller than the 80 kDa marker the expected sizes based on their sequence with a calculated mass of 72.03 kDa and thus probably represent the intact pro-form of AtCEP1 with the C-terminal KDEL-motif still attached, however in different conformation. This double band was already found for the analogous pro-3xHA-mCherry-AtCEP2-KDEL reporter protein (Hierl et al., 2013). The non-functional reporter protein pro-3xHA-EGFP-KDEL was also detectable from day 7 post inoculation on and was found until day 18 post inoculation (Figure 5).

### Cells attacked by *erysiphe cruciferarum* express EGFP-AtCEP1 in the endoplasmic reticulum and accumulate it around haustoria

**Figure 6:**
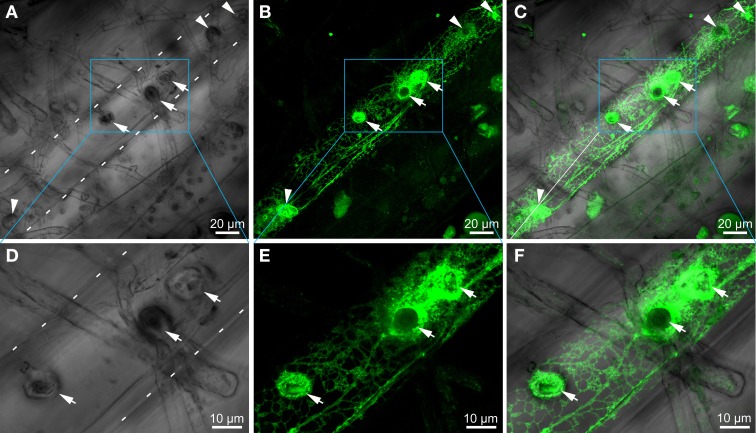
**Cells attacked by *Erysiphe cruciferarum* express EGFP-AtCEP1 in the endoplasmic reticulum and accumulate it around haustoria**. Localization of EGFP tagged AtCEP1 in *Erysiphe cruciferarum* attacked cells of *Arabidopsis* WT cells (*atcep1* mutant plants transformed with the functional reporter P_CEP1_::pre-pro-3xHA-EGFP-AtCEP1-KDEL) upon fungal leaf inoculation 12 dpi. Brick like epidermis cells close to the main vessel are shown; cell outlines are indicated by white dotted lines **(A,D)**. **(A)** Brightfield image of fungal structures on the leaf. Six bulbous haustoria are visible in the attacked cell (arrows and arrowheads). **(B)** EGFP-AtCEP1 shows localization in cortical net like structures and around haustoria. **(C)** Overlay of **(A,B)**. **(D–F)** Strong accumulation of EGFP-AtCEP1 can be observed around the haustoria. Pictures display a magnified optical section of the area marked by the blue box in the pictures **(A–C)** in the level of the intracellular haustoria. Pictures are maximum projections of 24 optical sections **(A–C)** or 12 optical sections **(D,E)**, respectively, with 0.5 μm increments.

To investigate the subcellular localization of AtCEP1 *in vivo*, we analyzed complemented mutant plants (*atcep1* knock out plants transformed with the functional reporter P_CEP1_::pre-pro-3xHA-EGFP-AtCEP1-KDEL) during interaction with *E. cruciferarum* (12 dpi) by CLSM (Figure 6). Without inoculation, we never detected the functional EGFP fusion of AtCEP1. 12 dpi, the EGFP-AtCEP1 fusion protein displayed localization within the entire endoplasmatic reticulum of cells that were successfully penetrated by the fungus and accumulated especially around established haustoria (Figures 6A–C). A strong labeling around haustoria was also observed at the haustorial plane, where the network of the endoplasmic reticulum seemed to be very dense (Figures 6D–F).

### Wild type plants exhibit significantly more dead epidermal cells and less established haustoria as compared to *atcep1* mutants upon inoculation with *erysiphe cruciferarum*

**Figure 7:**
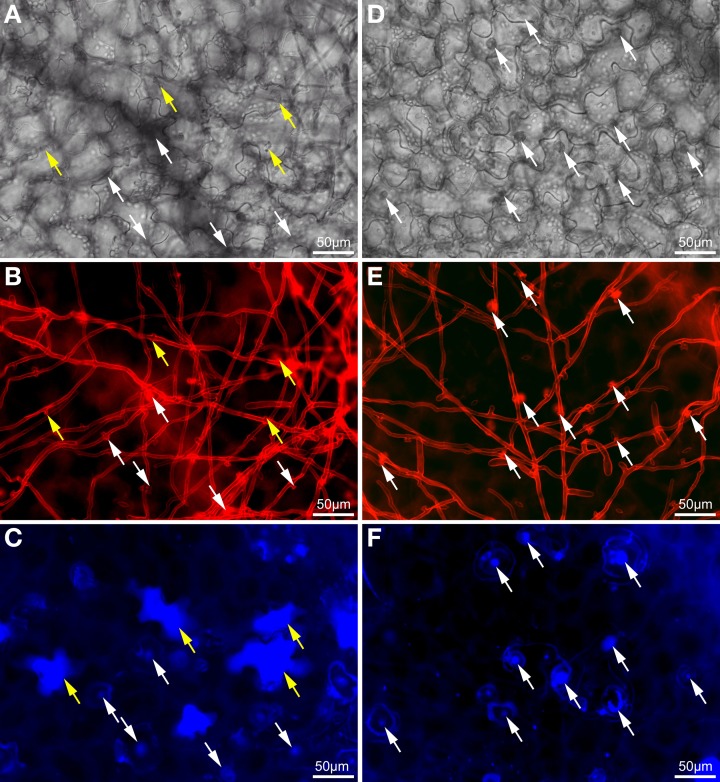
**Wild type plants exhibit significantly more dead epidermal cells and less established haustoria as compared to *atcep1* mutants upon inoculation with *Erysiphe cruciferarum***. Microscopic phenotype of powdery mildew attacked cells on *Arabidopsis* WT (Col0) and the *atcep1* knock out mutant (SAIL_158_B06) 5 dpi. WT cells show a frequent hypersensitive response (HR)-like reaction of penetrated cells **(A–C)** whereas no HR-like reaction is observed in the *atcep1* mutant **(D–F)**. **(A,D)** Brightfield image of the selected areas. **(B,E)** Fungal structures on the leaf surface and bulbous haustoria marked by white arrows were stained with wheat germ agglutinine-tetramethylrhodamine. **(C,F)** Callose was stained by methyl blue staining. Punctate methyl blue stained structures show papilla/cell wall appositions and encapsulated haustoria. Completely stained cells marked by yellow arrows indicate an HR-like cell death.

**Figure 8:**
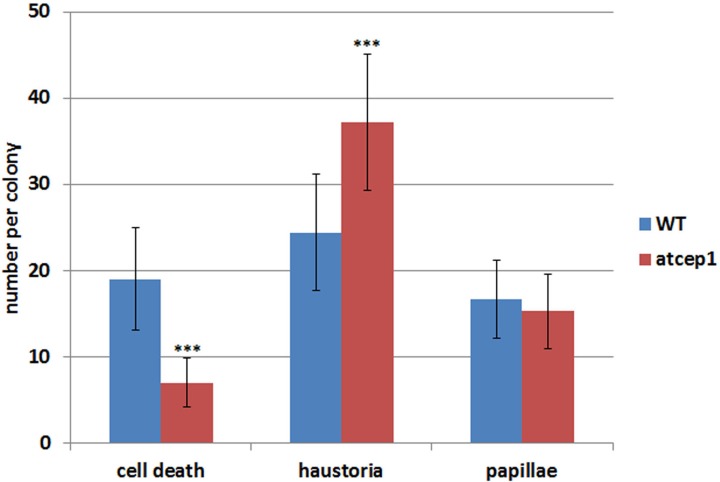
**The epidermal cell death is suppressed in cells attacked by *Erysiphe cruciferarum* on the *atcep1* mutant 5 dpi compared to the WT**. Columns show the frequency per colony of epidermal cell death, haustoria and papilla in cells attacked by *E. cruciferarum*. Data present the mean of 54 colonies on three leaves per wild type and *atcep1* mutant line, respectively. Error bars are standard deviations. Differences between wild type and *atcep1* are highly significant after two sided student's *t*-test *p* < 0.001 (^***^). The results were similarly reproduced in a second independent inoculation with 48 colonies on three leaves per WT and *atcep1* mutant line, respectively.

Visual examination of wild type and *atcep1* mutant plants upon infection with *E. cruciferarum* suggested a role for AtCEP1 in restricting the development of a biotrophic fungus (Figure 4, Supplemental Figure S3). To assess the *atcep1* knock out phenotype on a microscopic level we used two different labels: red fluorescent wheat germ agglutinin that stains the chitin of fungal hyphae and methyl blue that stains the callose at papillae and encasements of established haustoria as well as whole cells that die in the course of epidermal HR-like cell death (Figure 7). At 5dpi, fungal colonies on the wild type were grown to a size that about 24 haustoria were established per single fungal colony. In an average colony, 17 fungal attempts to penetrate were associated with localized callose depositions (papillae) without a visible haustorium. More than 19 cells per fungal colony displayed whole-cell callose deposition indicative of cell death, which was associated with fungal infection structures, in most cases collapsed haustoria. By contrast, the *atcep1* mutant displayed significantly less epidermal cell death (7 cells per colony) per fungal colony and at the same time supported the development of more haustoria (37 per colony) in living cells. Hence, the *atcep1* mutants displayed a failure to restrict establishment or maintenance of fungal haustoria under execution of an HR-like epidermal cell death (Figure 8).

### Pathogen-responsive genes expression in *atcep1*

**Figure 9:**
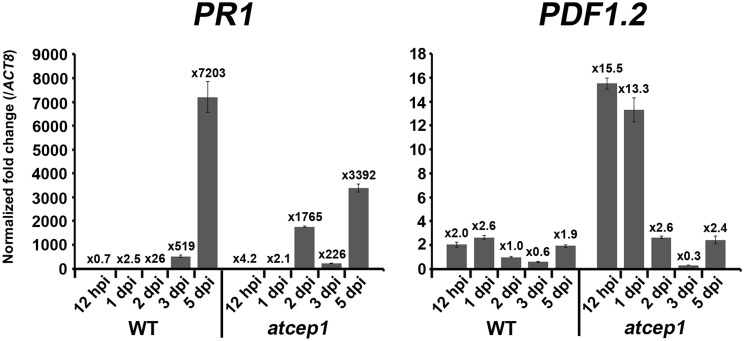
**Pathogen-responsive genes expression in *atcep1***. Relative gene expression of *PR1* and *PDF1.2* in wild type and *atcep1* plants 12 h post mildew inoculation (hpi) and 1, 2, 3, and 5 days post mildew inoculation (dpi). qRT-PCR was performed with *PR1* and *PDF1.2* gene-specific primers. Expression levels were normalized to the reference gene *ACT8* and expression before inoculation in each genotype was set to 1. The results were similarly reproduced by three independent inoculations. Error bars represent standard error of the mean (SE).

The difference in visual scoring leaf symptoms between wild type and *atcep1* mutant plants was most obvious at 9 to 13 dpi. However, differences were observed in fungal development and in plant cell death responses at the microscopic level already at 5 dpi. By contrast, no differences were observed in fungal development or host defense responses at 1 dpi (data not shown). We also tested wild type and *atcep1* mutants for differential expression of defense associated genes at 12 hpi, 1 dpi, 2 dpi, 3 dpi and 5 dpi. We measured relative expression of biotic stress markers *PR1* and *PDF1.2* (Reymond and Farmer, 1998) in wild type and *atcep1* plants after powdery mildew inoculation. Expression levels were normalized to the reference gene *ATC8*, and expression before inoculation in each genotype was set to 1. At distinct stages of the interaction, powdery mildew inoculation provoked very high induction of *PR1*. *PR1* gene expression was attenuated in super-susceptible *atcep1* mutants at 5 dpi, which was coinciding with lower levels of epidermal cell death at this time (Figures 8, 9). By contrast, *PDF1.2* expression in *atcep1* transiently exceeded wild type level at 0.5 dpi and at 1 dpi (Figure 9).

## Discussion

PCD is part of the HR, which restricts growth of biotrophic and hemibiotrophic pathogens in both basal resistance and in effector-triggered immunity (Dickman and Fluhr, 2013). In plants like in animals, proteases often mediate the decisive or executing steps of PCD. Papain-type cysteine proteases are involved in plant-microbe interactions. They are expressed in response to biotic stress and can be direct or indirect targets of microbial virulence effectors (Shindo and Van der Hoorn, 2008). This supports a fundamental function of papain-type proteases in plant defense. However, the identity and regulation of the proteases that are functional in particular plant-microbe interactions or in specific stages of such interactions are largely unknown. We show here that an ER-resident papain-type protease, which has been implicated in developmental cell death and tissue remodeling, is co-opted for a late defense reaction in a compatible interaction with a biotrophic fungus.

We tested the ability of wild type and *atcep1* mutants to restrict growth of the biotrophic fungus *E. cruciferarum* to find possible evidence for a role of AtCEP1 in biotic stress responses and for a role of late epidermal cell death in restricting parasitic growth of an adapted powdery mildew fungus. This revealed super-susceptibility of two independent *AtCEP1* T-DNA insertion mutants to powdery mildew. Both mutants lacked expression of *AtCEP1* and can thus be considered as loss-of-function mutants. Basal resistance of *Arabidopsis* was re-constituted by a functional but not by a non-functional complementation construct. A native promoter functional AtCEP1-reporter construct allowed for the detection of AtCEP1 protein accumulation during late stages of the interaction. Together, this suggests that AtCEP1 is involved in basal resistance of *Arabidopsis* to powdery mildew.

Little is known about the function and efficacy of plant defense responses in compatible plant-pathogen interactions. Late epidermal PCD, as observed here, could either be an indication of fungal failure to maintain compatibility at the single cell level for a long time or an effective basal defense response of the susceptible host. Our data suggest that this type of PCD is under genetic control of the host and partially restricts fungal development during late stages of the interaction and sporulation. It is generally believed that in a compatible interaction of a susceptible host with a virulent pathogen most plant immune responses are successfully suppressed by pathogen effector molecules. Indeed, host papain proteases, which function in defense against nematodes, fungi or oomycetes, are inhibited by host and pathogen protease inhibitors that are delivered during compatible interactions (Bozkurt et al., 2011; Lozano-Torres et al., 2012; van der Linde et al., 2012). Partial inhibition of AtCEP1 by a protease inhibitor from *E. cruciferarum* or the late accumulation of AtCEP1 protein during pathogen interaction would explain why the pathogenesis phenotype of *atcep1* was of quantitative nature. Furthermore, reporter expression of EGFP-AtCEP1 from P_CEP1_::pre-pro-3xHA-EGFP-AtCEP1-KDEL was detectable but irregular in cells penetrated by *E. cruciferarum.* Basal expression of the *AtCEP1* gene is also low but detectable in leaves but higher in seeds, siliques, generative tissues and parts of the root (Helm et al., 2008 and unpublished results of the authors). Late defense-related expression points to possible hormone regulation of AtCEP1 during pathogenesis-induced physiological perturbations rather than expression in response to pathogen-associated molecular patterns, which trigger early immune responses. Alternatively, pathogenesis might have caused a loss of tissue identity.

Cells attacked by *E. cruciferarum* expressed EGFP-AtCEP1 in the ER and accumulated it around haustoria but not in ricinosome-like structures or ER-bodies, in which AtCEP2 can be found (Hierl et al., 2012, 2013). Interestingly, mutants lacking functional AtCEP1 did not show the same frequency of cell death in attacked epidermal cells, when compared to wild type. This suggests that AtCEP1 is required to undergo this type of PCD during interaction with *E. cruciferarum*. However, we cannot yet distinguish whether this is due to a function in regulating epidermal PCD or in affecting the functionality of fungal haustoria. The accumulation of ER and EGFP-AtCEP1 at the plant-fungus interface let us speculate about a possible leakiness of the plant ER to the apoplast or to the extrahaustorial matrix. There are examples of plant KDEL-motif containing ER proteins, including papain-type proteases, that have a second destination in the apoplast or in the vacuole (Jones and Herman, 1993; Okamoto et al., 2003). Late-endosomal multivesicular compartments, which are by default delivered to the vacuole, and the tonoplast can also fuse with the plasma membrane during plant-pathogen interactions. Additionally, it is still unclear whether the host extrahaustorial membrane is an extension of the plasma membrane or originates from an endomembrane (An et al., 2007; Hatsugai et al., 2009; Hückelhoven and Panstruga, 2011; Dickman and Fluhr, 2013; Drakakaki and Dandekar, 2013). Hence, AtCEP1 could function in inhibiting haustorial functions when the protein leaks into the apoplast. Since KDEL CysEPs contain a cleavable KDEL ER retention motif (Figure 3), which is processed during protein activation (Than et al., 2004; Hierl et al., 2013), a novel mechanism would need to be postulated for controlled release of proteins from the ER.

Haustoria are likely the fungal cells, from which effector proteins are released to suppress host immune responses including the PCD. Therefore late expression of AtCEP1 could restrict haustorial functions in suppression of cell death. This is further supported because the reduction of cell death events in *atcep1* was accompanied by a complementary increase in haustoria per fungal colony. Indeed, most of the dead cells in the wild type were successfully penetrated and contained haustoria. Alternatively, AtCEP1 might directly function in epidermal PCD. KDEL-cysteine peptidases like AtCEP1 are considered as late-acting proteases that digest cell wall proteins during the final stages of PCD and tissue remodeling after cellular disintegration (Helm et al., 2008; Hierl et al., 2012). However, a sole post cell death digestive function of AtCEP1 would not explain restriction of fungal growth. Post-cell death tissue-clearance cannot determine the outcome of the interaction, because the fungus strictly depends on an intact host cell for biotrophy at the single-cell level. An alternative explanation for the phenotype would be a new function of AtCEP1 in the initiation of epidermal cell death.

Epidermal PCD occurred late in the interaction with the virulent powdery mildew fungus. This makes it different from a canonical HR, which is observed early in effector-triggered immunity and is based on specific nonself recognition. We do not yet know whether this type of late epidermal cell death mechanistically resembles HR or a novel type of PCD. Additionally, defense gene expression was altered in *atcep1* mutants before the pathogenesis-related phenotype became detectable. This might hind at a function of AtCEP1 in communication of the ER with the nucleus in stress-related gene expression.

## Conclusion

Our data suggest a new function of AtCEP1 in late epidermal PCD in the interaction with the powdery mildew fungus. This PCD might possibly be a new type of PCD rather than classical HR. Future studies will shed light on the nature and regulation of this type of PCD and its role in interaction with other pathogens. Additionally, early differential expression of stress markers in *atcep1* points to a possible involvement of AtCEP1 in crosstalk between the ER and biotic stress responses, that takes place before cell death is observed. Apparently, the ER KDEL-peptidase AtCEP1, which otherwise may function in developmental PCD, has an additional and pivotal function in translation of pathogenesis-related stress into leaf epidermal cell death and pathogen defense.

### Conflict of interest statement

The authors declare that the research was conducted in the absence of any commercial or financial relationships that could be construed as a potential conflict of interest.

## Abbreviations

CLSM: confocal laser scanning microscopy
EGFP: enhanced green fluorescent protein
3xHA: three-fold hemagglutinin (HA) tag
HR: hypersensitive response.
