# Supplementary material for: Endoplasmic reticulum KDEL-tailed cysteine endopeptidase 1 of Arabidopsis (AtCEP1) is involved in pathogen defense
Source: Front Plant Sci. 2014 Feb 24;5:58. doi: 10.3389/fpls.2014.00058 (PMC3932416; doi:10.3389/fpls.2014.00058)
Supplement: Supplementary file 1 [file DataSheet1.PDF]

Figure S1: Cloning strategy in pGreen 0029 for expression of the fusion protein pre-pro-3xHA-EGFP-AtCEP1-KDELunder control of the endogenous AtCEP1 promoter.

DNA sequence:

GGTACCaacgaatacatttatttttctgaaacaagatggattatcggttacagatttagaagcaaacgcaattc
atgacactgcatcctctttgtagtgatgtgatgtgcgtgttagctgtgtgtgtccgcgtttatcttttttttttt
tgttacttttctcctaacttagttgttattacgtgcttttcccaatatcaagttaaatcacacacacttcccacc
accttccttcattttctttccgaaatttagcatttagtcctcttttgacaaatagtatcttctttcttaaattt
aagatctttctttagtctttagacaacagttattgtaatgtcttttagttgaatgacataagttaggccaaaata
atttgagttcaattttcaaaagcgttgatgaaagctaaaaccaatgactaaaaatgacatctaatcggctcctga
aaaccataatcagatatcatatgacaaatactaaaccaaaatttttacctttcaaataaaataatatttgcagat
agatttaaaatagtaaaggttattgaaaaaaaaatacaatagaaaaacataatattacctaatatttattacatt
ttaaaagatatatagaaaaacaaaatcttagtttcgacgatggaattttttttttttatatagaacatcacatacc
aaataactttagttctcttattgtgaaaacaatggaagaaattgactttttaaaaataattttgaaattgatttc
tttgttcacataccaaacaacttcgttttattatcgtgaaaaaaagataacgtaaatggttagccacacatgcac
ggcttttccaaattatcataaggatccatatttgtaaaagaaaaaaatacataatccacataattaatcatagtg
aggttacatgaataacagagttcctagctagtaaagataaattgttgcgtacaagttgtgctttgattaattatc
actatacgtgcttcatataaaaatgtcattttacacttatatatattaagtcatctcatcgccgactatttccca
aaatcatcaattattgttaccaagaataagaaaagtaaaatgagagagcttgcttgcgccattaaatcaacataa
agttcaattatagtttgaaatctttatgaatctccattaattaatgtatccatgtcactaaccttgaaatctttg
ttgaatcccacaagtaatcctttaaccccttttggtacttttcattacatatcgtcatcacaataacctctcacc
aatgtatctataatccctatataaacactttctctttagtcctgattaccatcaacaaactcaatcacaatcaca
agtaaatacaaaaatcaccatagaaaaat**ATG**AAGCGATTTATTGTTCTTGCGCTTTGCATGCTTATGGTTCTTG
AAACCACAAAGGGTTTAGATTTCCATAACAAAGATGTGGAATCAGAGAATAGCTTGTGGGAGCTATACGAACGGT
GGAGGAGTCACCACACTGTTGCTAGGAGCCTTGAAGAGAAAGCTAAGAGGTTCAACGTGTTCAAGCACAACGTGA
AGCACATCCATGAGACCAACAAGAAGGATAAATCTTACAAGCTCAAGCTAAACAAGTTTGGTGACATGACTAGTG
AAGAATTTAGGAGAACATACGCTGGTTCAAACATCAAACATCATAGGATGTTTCAAGGCGAAAAAAAGGCCACAA
AGAGCTTCATGTATGCAAATGTAAATACTCTCCCAACCCTCGAGtacccatacgacgttcctgactatgcgtcac
tctacccctatgacgtaccggattatgcatccctatatccgtatgatgttccagattacgcttctctacgttcct
ctagaggcgtccaccatatgGTCGACATGGTGAGCAAGGGCGAGGAGCTGTTCACCGGGGTGGTGCCCATCCTGG
TCGAGCTGGACGGCGACGTAAACGGCCACAAGTTCAGCGTGTCCGGCGAGGGCGAGGGCGATGCCACCTACGGCA
AGCTGACCCTGAAGTTCATCTGCACCACCGGCAAGCTGCCCGTGCCCTGGCCCACCCTCGTGACCACCCTGACCT
ACGGCGTGCAGTGCTTCAGCCGCTACCCCGACCACATGAAGCAGCACGACTTCTTCAAGTCCGCCATGCCCGAAG
GCTACGTCCAGGAGCGCACCATCTTCTTCAAGGACGACGGCAACTACAAGACCCGCGCCGAGGTGAAGTTCGAGG
GCGACACCCTGGTGAACCGCATCGAGCTGAAGGGCATCGACTTCAAGGAGGACGGCAACATCCTGGGGCACAAGC
TGGAGTACAACTACAACAGCCACAACGTCTATATCATGGCCGACAAGCAGAAGAACGGCATCAAGGTGAACTTCA
AGATCCGCCACAACATCGAGGACGGCAGCGTGCAGCTCGCCGACCACTACCAGCAGAACACCCCCATCGGCGACG
GCCCCGTGCTGCTGCCCGACAACCACTACCTGAGCACCCAGTCCGCCCTGAGCAAAGACCCCAACGAGAAGCGCG
ATCACATGGTCCTGCTGGAGTTCGTGACCGCCGCCGGGATCACTCTCGGCATGGACGAGCTGTACAAGTCCGGAG
CTGCTGCCGCTGCCGCTGCGGCAGCGGCCGGATCCCTCCCAACCTCCGTTGATTGGAGAAAGAATGGAGCCGTCA
CTCCTGTCAAAAACCAAGGCCAATGCGGGAGTTGTTGGGCGTTTTCAACAGTTGTTGCGGTGGAAGGATCAACC
AAATAAGAACTAAAAAGCTGACATCACTCTCAGAGCAAGAGCTAGTAGATTGTGATACAAACCAGAACCAAGGAT
GCAATGGAGGTCTAATGGACCTTGCTTTTGAGTTCATCAAGGAGAAAGGAGGACTCACAAGTGAGCTAGTGTACC
CTTACAAGGCTTCTGATGAAACTTGTGACACAAACAAAGAAAATGCTCCGGTAGTTTCAATCGATGGACACGAAG
ATGTTCCTAAGAACAGCGAGGATGATCTAATGAAAGCTGTTGCTAATCAGCCTGTTTCTGTTGCTATTGATGCTG
GAGGCTCAGACTTTCAATTCTACTCCGAGGGAGTGTTTACCGGGAGATGCGGAACAGAGCTAAACCATGGAGTTG
CGGTAGTAGGGTATGGAACAACGATAGACGGAACAAAGTATTGGATTGTTAAGAATTCATGGGGAGAGGAATGGG
GAGAGAAAGGATACATAAGAATGCAGAGAGGGATTCGTCATAAAGAAGGACTTTGTGGTATTGCAATGGAAGCTT
CTTATCCTCTCAAGAACTCCAACACTAACCCTTCTAGACTTTCCTTGGACTCGCTTAAGGATGAACTC**TAA**atta
aatctctttatattaagattaaatagacattgacaagtttgtttatgatgattgtttttttttttaaatatattt
ctttttcattaataggttatggttttcaatgtatgaagttgatgggaatcgtttgaattcgttaaatgaataaat
aaatggttttatgtGCGGCCGC

Primer used for PCR amplification and cloning:

Primer pair 1 for amplification of the promoter and the pre-pro-sequence (1763 bp):
Sense: gtcGGTACCaacgaatacatttattttc
Antisense: ctcCTCGAGggttgggagagtatttaca

Primer pair 2 for amplification of the 3xHA tag (132 bp):
Sense: aaaCTCGAGtacccatacgacgttcctg
Antisense: catGTCGACcatatggtggacgcctct

Primer pair 3 for amplification of EGFP (759 bp):
Sense: gttcctGTCGAC**atg**gtgagcaagggcgagg
Antisense: cctgaaGGATCCggccgctgccgcagcggc

Primer pair 4 for amplification of AtCEP1-KDEL-3'UTR (885 bp):
Sense: aagGGATCCctcccaacctccgttgat
Antisense: gtgGCGGCCGCacataaaaccatttatttattc

Protein-Sequence:
MKRFIVLALCMLMVLETTKGLDFHNKDVESENSLWELYERWRSHHTVARSLEEKAKRFNVFKHNVKHIHETNKKD
KSYKLKLNKFGDMTSEEFRRTYAGSNIKHHRMFQGEKKATKSFMYANVNTLPTLEYPYDVPDYASLYPYDVPDYA
SLYPYDVPDYASLRSSRGVHHMVDMVSKGEELFTGVVPILVELDGDVNGHKFSVSGEGEGDATYGKLTLKFICTT
GKLPVPWPTLVTTLTYGVQCFSRYPDHMKQHDFFKSAMPEGYVQERTIFFKDDGNYKTRAEVKFEGDTLVNRIEL
KGIDFKEDGNILGHKLEYNYNSHNVYIMADKQKNGIKVNFKIRHNIEDGSVQLADHYQQNTPIGDGPVLLPDNHY
LSTQSALSKDPNEKRDHMVLLEFVTAAGITLGMDELYKSGAAAAAAAAAAGSLPTSVDWRKNGAVTPVKNQGQCG
SCWAFSTVVAVEGINQIRTKKLTSLSEQELVDCDTNQNQGCNGGLMDLAFEFIKEKGGLTSELVYPYKASDETCD
TNKENAPVVSIDGHEDVPKNSEDDLMKAVANQPVSVAIDAGGSDFQFYSEGVFTGRCGTELNHGVAVVGYGTTID
GTKYWIVKNSWGEEWGEKGYIRMQRGIRHKEGLCGIAMEASYPLKNSNTNPSRLSLDSLKDEL

Pre - Pro - 3xHA - EGFP - AtCEP1 - KDEL
Amino acids in white were introduced due to cloning strategy

Figure S2: Cloning strategy in pGreen 0029 for expression of the fusion protein pre-pro-3xHA-EGFP-KDELunder control of the endogenous AtCEP1 promoter.

DNA sequence:

GGTACCaacgaatacatttatttttctgaaacaagatggattatcggttacagatttagaagcaaacgcaattc
atgacactgcatcctctttgtagtgatgtgatgtgcgtgttagctgtgtgtgtccgcgtttatcttttttttttt
tgttacttttctcctaacttagttgttattacgtgcttttcccaatatcaagttaaatcacacacacttcccacc
accttccttcatttctttccgaaatttagcattttagtcctcttttgacaaatagtatcttctttcttaaattt
aagatctttctttagtctttagacaacagttattgtaatgtcttttagttgaatgacataagttaggccaaaata
atttgagttcaattttcaaaagcgttgatgaaagctaaaaccaatgactaaaaatgacatctaatcggctcctga
aaaccataatcagatatcatatgacaaatactaaaccaaaatttttacctttcaaataaaataatatttgcagat
agatttaaaatagtaaaggttattgaaaaaaaaatacaatagaaaaacataatattacctaatatttattacatt
ttaaaagatatatagaaaaacaaaatcttagtttcgacgatggaattttttttttttatatagaacatcacatacc
aaataactttagttctcttattgtgaaaacaatggaagaaattgacttttaaaaataattttgaaattgatttc
tttgttcacataccaaacaacttcgttttattatcgtgaaaaaaagataacgtaaatggttagccacacatgcac
ggcttttccaaattatcataaggatccatatttgtaaaagaaaaaaatacataatccacataattaatcatagtg
aggttacatgaataacagagttcctagctagtaaagataaattgttgcgtacaagttgtgctttgattaattatc
actatacgtgcttcatataaaaatgtcattttacacttatatatattaagtcatctcatcgccgactatttccca
aaatcatcaattattgttaccaagaataagaaaagtaaaatgagagagcttgcttgcgccattaaatcaacataa
agttcaattatagtttgaaatctttatgaatctccattaattaatgtatccatgtcactaaccttgaaatctttg
ttgaatcccacaagtaatcctttaaccccttttggtacttttcattacatatcgtcatcacaataacctctcacc
aatgtatctataatccctatataaacactttctctttagtcctgattaccatcaacaaactcaatcacaatcaca
agtaaatacaaaaatcaccatagaaaaat**ATG**AAGCGATTTATTGTTCTTGCGCTTTGCATGCTTATGGTTCTTG
AAACCACAAAGGGTTTAGATTTCCATAACAAAGATGTGGAATCAGAGAATAGCTTGTGGGAGCTATACGAACGGT
GGAGGAGTCACCACACTGTTGCTAGGAGCCTTGAAGAGAAAGCTAAGAGGTTCAACGTGTTCAAGCACAACGTGA
AGCACATCCATGAGACCAACAAGAAGGATAAATCTTACAAGCTCAAGCTAAACAAGTTTGGTGACATGACTAGTG
AAGAATTTAGGAGAACATACGCTGGTTCAAACATCAAACATCATAGGATGTTTCAAGGCGAAAAAAAGGCCACAA
AGAGCTTCATGTATGCAAATGTAAATACTCTCCCAACCCTCGAGtacccatacgacgttcctgactatgcgtcac
tctacccctatgacgtaccggattatgcatccctatatccgtatgatgttccagattacgcttctctacgttcct
ctagaggcgtccaccatatgGTCGACATGGTGAGCAAGGGCGAGGAGCTGTTCACCGGGGTGGTGCCCATCCTGG
TCGAGCTGGACGGCGACGTAAACGGCCACAAGTTCAGCGTGTCCGGCGAGGGCGAGGGCGATGCCACCTACGGCA
AGCTGACCCTGAAGTTCATCTGCACCACCGGCAAGCTGCCCGTGCCCTGGCCCACCCTCGTGACCACCCTGACCT
ACGGCGTGCAGTGCTTCAGCCGCTACCCCGACCACATGAAGCAGCACGACTTCTTCAAGTCCGCCATGCCCGAAG
GCTACGTCCAGGAGCGCACCATCTTCTTCAAGGACGACGGCAACTACAAGACCCGCGCCGAGGTGAAGTTCGAGG
GCGACACCCTGGTGAACCGCATCGAGCTGAAGGGCATCGACTTCAAGGAGGACGGCAACATCCTGGGGCACAAGC
TGGAGTACAACTACAACAGCCACAACGTCTATATCATGGCCGACAAGCAGAAGAACGGCATCAAGGTGAACTTCA
AGATCCGCCACAACATCGAGGACGGCAGCGTGCAGCTCGCCGACCACTACCAGCAGAACACCCCCATCGGCGACG
GCCCCGTGCTGCTGCCCGACAACCACTACCTGAGCACCCAGTCCGCCCTGAGCAAAGACCCCAACGAGAAGCGCG
ATCACATGGTCCTGCTGGAGTTCGTGACCGCCGCCGGGATCACTCTCGGCATGGACGAGCTGTACAAGTCCGGAG
CTGCTGCCGCTGCCGCTGCGGCAGCGGCCGGATCCTTGGACTCGCTTAAGGATGAACTC**TAA**attaaatctcttt
atattaagattaaatagacattgacaagtttgtttatgatgattgtttttttttttaaatatatttctttttcat
taataggttatggttttcaatgtatgaagttgatgggaatcgtttgaattcgttaaatgaataaataaatggttt
tatgtGCGGCCGC

Primer used for PCR amplification and cloning:

Primer pair 1 for amplification of the promoter and the pre-pro-sequence (1763 bp):

Sense: gtcGGTACCaacgaatacatttatttttc

Antisense: ctcCTCGAGggttgggagagtatttaca

Primer pair 2 for amplification of the 3xHA tag (132 bp):

Sense: aaaCTCGAGtacccatacgacgttcctg

Antisense: catGTCGACcatatggtggacgcctct

Primer pair 3 for amplification of EGFP (759 bp):

Sense: gttcctGTCGAC**atg**gtgagcaagggcgagg

Antisense: cctgaaGGATCCggccgctgccgcagcggc

Primer pair 4 for amplification of AtCEP1-KDEL-3'UTR (201 bp):

Sense: aagGGATCCttggactcgcttaaggatg

Antisense: gtgGCGGCCGCacataaaaccatttatttattc

Protein-Sequence:

MKRFIVLALCMLMVLETTKGLDFHNKDVESENSLWELYERWRSHHTVARSLEEKAKRFNVFKHNVKHIHETNKKD
KSYKLKLNKFGDMTSEEFRRTYAGSNIKHHRMFQGEKKATKSFMYANVNTLPTLEYPYDVPDYASLYPYDVPDYA
SLYPYDVPDYASLRSSRGVHHMVDMVSKGEELFTGVVPILVELDGDVNGHKFSVSGEGEGDATYGKLTLKFICTT
GKLPVPWPTLVTTLTYGVQCFSRYPDHMKQHDFFKSAMPEGYVQERTIFFKDDGNYKTRAEVKFEGDTLVNRIEL
KGIDFKEDGNILGHKLEYNYNSHNVYIMADKQKNGIKVNFKIRHNIEDGSVQLADHYQQNTPIGDGPVLLPDNHY
LSTQSALSKDPNEKRDHMVLLEFVTAAGITLGMDELYKSGAAAAAAAAAAGSLLDSLKDEL

Pre – Pro – 3xHA – EGFP - KDEL

Amino acids in white were introduced due to cloning strategy

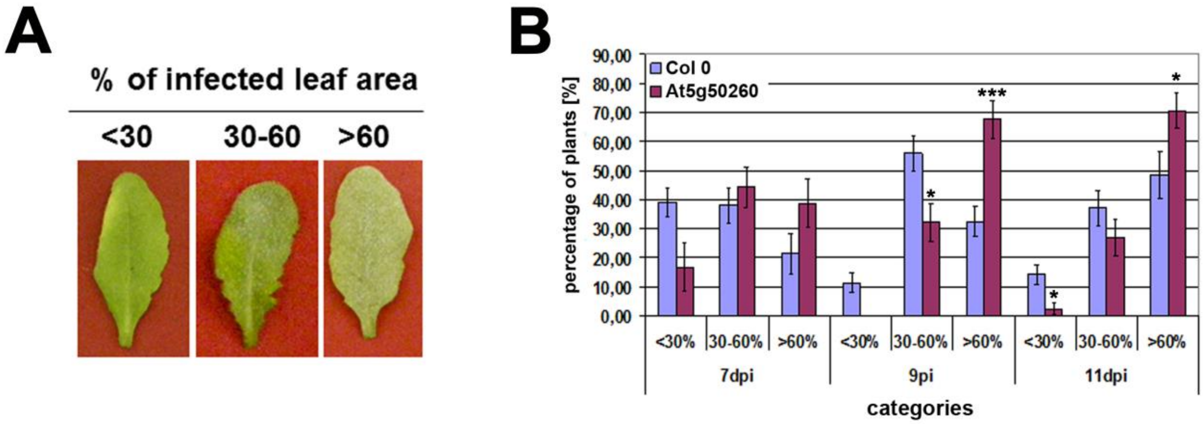

Figure S3. Disease symptoms of wild type (Col0) and *atcep1* mutant plants (SALK_013036, At5g50260) upon infection with *E. cruciferarum* spores. Disease symptoms were scored by visual examination of the whole plant 7, 9 and 11 days after inoculation (dpi). Infected leaves were distributed in the three categories <30%, 30-60%, and >60% diseased leaf area. A. Representative leaves were excised and photographed 11 dpi. B. Columns represent the frequency of plants distributed in the three categories of susceptibility. Differences between wild type and *atcep1* are highly significant (***) after two sided student's t-test, $p<0.05$ (*), $p<0.001$ (***). Each experiment comprised 8-10 plants per line. Data represent the respective means of five experiments from independent inoculation events. Error bars represent standard error of the mean (SE).
